# Obstetric professionals’ perceptions of non-invasive prenatal testing for Down syndrome: clinical usefulness compared with existing tests and ethical implications

**DOI:** 10.1186/s12884-017-1474-6

**Published:** 2017-09-05

**Authors:** Olivia Miu Yung Ngan, Huso Yi, Samuel Yeung Shan Wong, Daljit Sahota, Shenaz Ahmed

**Affiliations:** 1 JC School of Public Health and Primary Care, Faculty of Medicine, The Chinese University of Hong Kong, Shatin, New Territories, Hong Kong SAR China; 2 CUHK Centre for Bioethics, Faculty of Medicine, The Chinese University of Hong Kong, Shatin, New Territories, Hong Kong SAR China; 3 Department of Obstetrics and Gynaecology, Faculty of Medicine, The Chinese University of Hong Kong, Shatin, New Territories, Hong Kong SAR China; 4 Division of Public Health and Primary Care, Leeds Institute of Health Sciences, University of Leeds, Leeds, UK

**Keywords:** Non-invasive prenatal test, Cell-free fetal DNA, Clinical decision-making, Attitude, Ethical concern, Informed consent, Down syndrome, Hong Kong

## Abstract

**Background:**

While non-invasive prenatal testing (NIPT) for fetal aneuploidy is commercially available in many countries, little is known about how obstetric professionals in non-Western populations perceive the clinical usefulness of NIPT in comparison with existing first-trimester combined screening (FTS) for Down syndrome (DS) or invasive prenatal diagnosis (IPD), or perceptions of their ethical concerns arising from the use of NIPT.

**Methods:**

A cross-sectional survey among 327 obstetric professionals (237 midwives, 90 obstetricians) in Hong Kong.

**Results:**

Compared to FTS, NIPT was believed to: provide more psychological benefits and enable earlier consideration of termination of pregnancy. Compared to IPD, NIPT was believed to: provide less psychological stress for high-risk women and more psychological assurance for low-risk women, and offer an advantage to detect chromosomal abnormalities earlier. Significant differences in perceived clinical usefulness were found by profession and healthcare sector: (1) obstetricians reported more certain views towards the usefulness of NIPT than midwives and (2) professionals in the public sector perceived less usefulness of NIPT than the private sector. Beliefs about earlier detection of DS using NIPT were associated with ethical concerns about increasing abortion. Participants believing that NIPT provided psychological assurance among low-risk women were less likely to be concerned about ethical issues relating to informed decision-making and pre-test consultation for NIPT.

**Conclusions:**

Our findings suggest the need for political debate initially on how to ensure pregnant women accessing public services are informed about commercially available more advanced technology, but also on the potential implementation of NIPT within public services to improve access and equity to DS screening services.

## Background

Non-invasive prenatal testing (NIPT) for Down syndrome (DS) using cell-free DNA in maternal plasma (cff-DNA) is now commercially established [1–3], and available in many countries [4]. NIPT is considered a highly accurate screening test for DS as it has a sensitivity rate of 99.2% and false-positive rate (FPR) of 0.09% [5], compared with conventional first-trimester combined screening (FTS,≈ 90%, and 3.4%–5.4% respectively) [6]. Therefore, using NIPT reduces the need for invasive prenatal diagnostic tests (IPD - chorionic villus sampling (CVS) or amniocentesis), which carry a procedure risk of miscarriage ranging from 0.1–0.2% [7]. NIPT is not considered a diagnostic test because it has a positive predictive value (PPV) of less than 100% [6], occasionally giving non-reportable or false-positive/negative results [8, 9]. Therefore, it is considered to be a screening test, where a positive result requires confirmatory diagnostic testing [10, 11]. Like other countries in much of the world, antenatal testing is a routine part of pregnancy care in Hong Kong (HK), where every pregnant woman is offered blood tests and ultrasounds to evaluate the fetus’ conditions. Women at increased risk after screening (adjusted term risk of 1 in 250 or higher) are counselled regarding their available options, including a free of charge IPD in public hospitals or self-financed IPD or NIPT from private obstetricians.

It is well recognised that in addition to the clinical validity of NIPT, its implementation into public maternity services and the development of national/local guidelines should be based on key stakeholders’ views, particularly ethical, legal and social implication (ELSI) [12]. There is much research on stakeholders’ views [13, 14], generally showing that health professionals have a positive attitude towards implementing NIPT, but also some important ethical concerns and diverse views about how it should be implemented. For example, an American study among obstetricians reported diverse views about whether NIPT should be implemented as a primary or secondary screening test, and whether it should be offered to all or high-risk women [15].

Perception of implementing NIPT in public maternity services is likely to be context-specific [16]. For example, there has been a trend for rising maternal age at childbirth in HK*.* The local population-wide census reports that 70% of married women had their first baby during the first 3 years of marriage, with 28.9 as the median age of first marriage, and additionally reports one of the lowest fertility rates in the world at 1.04 per women [17]. Parents in HK prefer to have one or two children at most, partly for economic reasons [18], and regard having a baby with chromosomal abnormalities to be a significantly more negative lifetime event than a procedure-related loss of an unborn baby [19, 20]. Accordingly, local studies reported high rates of DS screening uptake of 96.7% [20–22] and termination of 93.5% (29/31), where 29 out of 31 women with a pregnancy with chromosomal abnormalities, confirmed by karyotyping, decided to undergo termination of pregnancy (TOP); the remaining three cases underwent spontaneous miscarriage [22]. Similar to other studies [23], parents in HK are highly likely to want reassurance that their unborn baby will not have preventable genetic conditions. Obstetric professionals in HK are likely to be aware of these trends in fertility and societal preferences for a ‘normal’ baby and, therefore, feel more pressure to ensure that women are offered the most appropriate technology as early as possible in pregnancy.

Most of the research on different stakeholders’ views of NIPT, however, has been conducted in Western populations, and the views of non-Western stakeholders are rarely sought. The aim of the present study was to explore the views of obstetric professionals in HK about (1) the clinical usefulness of NIPT in comparison with current DS screening and diagnostic practice, and (2) ethical concerns likely to arise from the use of NIPT.

## Methods

### Study settings

In HK, a 1st or 2nd trimester DS screening test has been offered to pregnant women in public hospitals (hence free of charge) since July 2010. Women can also opt for a range of prenatal tests privately. Overall, women are free to choose public and/or private sector services. Women considering screening at public and private institutions are provided with information about DS screening (e.g., pamphlets and videos), but are not ‘counselled’. Women identified at increased risk following screening (adjusted term risk of 1 in 250 or higher) in public services are counselled about subsequent options and informed about the availability of a free of charge IPD at the public hospital, and privately available IPD and NIPT [24].

### Recruitment of participants

Obstetricians, nurses and midwives providing FTS for DS in HK at the time of the study, practising in the public and/or private sector, were recruited during October 2013 to January 2014. All obstetricians listed on the HK Medical Council Registry were sent the study information sheet and questionnaire by post. Two reminders were sent to non-respondents at the three-week interval after the initial mailing. The response rate for the obstetricians was 27.2% (90/331). Midwives and nurses were recruited at hospitals (*n* = 15; 4 public and 11 private) and private clinics (*n* = 132) after obtaining approval from the respective departmental head of each institution. A written informed consent was obtained. A total of 279 surveys were delivered and 237 were collected, giving a response rate of 237/279 (84.9%). The overall response rate for the study was 53.6% (327/610). Ethical approval was granted by the University ethics committee.

### Data collection

A self-completion questionnaire was developed by a multidisciplinary group of clinicians and social scientists, based on a review of the ELSI literature on NIPT. The questionnaire was piloted with 5 obstetricians and 5 midwives to check content validity and preliminary psychometric properties. Based on the results of this pilot, minor changes were made before distribution. The questionnaire started with a brief description of NIPT, stating that NIPT had been available commercially in HK since December 2011 to detect chromosome aneuploidy (e.g. Down syndrome) from 10 weeks of gestation, with a sensitivity and specificity rate of over 99% and false-positive rate of 0.1%. The questionnaire then consisted of two parts to explore participants’ views about NIPT.

Part one covered respondents’ perceptions of the clinical usefulness of NIPT. Respondents were asked to compare NIPT with (a) current DS screening (FTS) and then (b) invasive prenatal diagnostic (IPD) tests by indicating their agreement, disagreement or uncertainty (ticking ‘yes’, ‘no’ or ‘don’t know’, respectively) for 6 descriptive statements about NIPT that were the most frequently mentioned NIPT test characteristics and advantages among stakeholders in Western studies. An additional statement was added in the IPD section to assess perceived procedural safety. The reliabilities of the measure in the study sample were assessed by internal consistency, that is correlations between items, and found be to be very good (Cronbach alpha = .84 for FTS and alpha = .86 for IPD). Part two covered perceptions of the ethical concerns likely to arise from the use of NIPT, using a 5-point Likert scale (1 = not at all concerned to 5 = extremely concerned), with 12 items categorized into four domains: (1) physician competency (two items; ‘lack of prior consultation about how NIPT is provided’ and ‘NIPT performed when physicians have inadequate knowledge’); (2) informed choice (two items; ‘women undertake NIPT without careful informed choice’ and ‘NIPT is performed when pregnant women have inadequate information’); (3) accessibility (four items; ‘NIPT will develop into mass routine screening’, ‘unnecessary prenatal testing performed among low-risk women’, ‘more pregnant women would undergo multiple screening tests’, and ‘unequal access to NIPT due to higher out-of-pocket expense’); and (4) social implications (four items; ‘NIPT can lead to discrimination against newborns with DS’, ‘abortion would become more prevalent’, ‘patients will have limited autonomy to have a baby with DS’ and ‘NIPT will develop in favor of non-medical purposes’). The measure was reliable (Cronbach alpha = .89).

### Data analysis

Chi-square and t-test were used to compare group differences. Kruskal-Wallis test was used to compare the relationship between the measures of ‘*clinical usefulness*’ and ‘*ethical concerns*’. To derive stable regression models with appropriate distributions, socio-demographic variables of education and religious affiliation were collapsed into dichotomous variables and the 5-point scale (1 = not at all concerned, 2 = slightly concerned, 3 = somewhat concerned, 4 = moderately concerned, 5 = extremely concerned) was regrouped into 3 categories (1 = not at all concerned, 2 = slightly-somewhat concerned, 3 = moderately-extremely concerned). Multiple ordinal logistic regressions were used to examine independent predictors for ‘*ethical concerns*’ controlling for the factors significant at *p* < 0.2 in univariate analyses including socio-demographic variables. Adjusted odds ratios (AORs) with 95% confidence intervals (CIs) were reported. AORs for factors related to ‘*clinical usefulness*’ were determined about respondents who indicated they were ‘uncertain.’

## Results

### Sample characteristics

Three hundred and 27 respondents completed the questionnaire. Their age ranged from 20 to 78 years (M = 39.1, SD = 12.1) and clinical experience ranged from 1 to 55 years (M = 12.5, SD = 11.1). Most respondents were female (86%) and had completed university-level or above education (79%). Twenty-eight percent (*n* = 90) of the respondents were obstetricians and 72% (*n* = 237) were midwives/nurses. Forty-two percent of the respondents (*n* = 137) worked in public hospitals. About one-third of respondents described themselves as Christian, 6% as Buddhists and 58% as having no religious affiliation. There was no significant difference between social-demographic and professional characteristics except that males were more likely than females to be obstetricians (χ^2^ = 86.7, *p* < 0.001) and have completed university education (χ^2^ = 5.34, *p* = 0.031).

### Perceptions of NIPT in comparison with first-trimester combined screening (FTS)

**Fig. 1:**
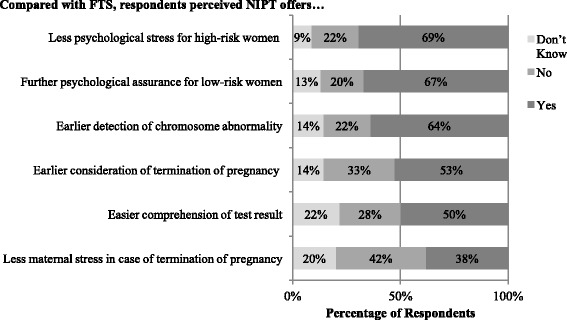
Comparison of Perceived Clinical Utility of NIPT with First-trimester Combined Screening (FTS)

Figure 1 shows respondents’ perceived clinical usefulness of NIPT compared with FTS. Respondents believed NIPT was likely to result in less psychological stress for high-risk women (69%) and more psychological assurance for low-risk women (67%). About 64% considered the availability of the test as early as the first-trimester was considered a favourable quality of NIPT. About half of the respondents believed that NIPT results were easier to understand (50%) and that NIPT enabled earlier consideration of TOP (53%). While 38% of the respondents believed NIPT resulted in less maternal stress in case of TOP, most respondents were unsure (42%).

Overall, the responses show that obstetricians had more formed views on the usefulness of NIPT compared with FTS (see Table 1). For example, their response of “don’t know” to the items ranged from 3% to 9% (median = 5%, M = 5%, SD = 2%), compared to midwives’ that ranged from 11% to 28% (median = 19%, M = 20%, SD = 5.6%). Furthermore, significant differences were found between respondents by profession and healthcare sector, but not education. Professionals working in public hospitals were more likely than those in private hospitals to believe that NIPT was as useful as FTS on psychological benefits in terms of reduction of maternal stress relating to pregnancy.

**Table 1:** Comparisons of Perceived Clinical Utility of NIPT with FTS by Demographic Characteristics

		Total (*n* = 327)	High school (*n* = 70)	University (*n* = 257)	χ^2^	Obstetrician (*n* = 90)	Midwife (*n* = 237)	χ^2^	Public Sector (*n* = 136)	Private Sector (*n* = 191)	χ^2^
Less physiological stress for high-risk women	Y	69%	69%	70%	5.80	68%	70%	4.85	61%	75%	7.24*
	N	22%	15%	23%		28%	19%		29%	17%	
	DK	9%	16%	7%		4%	11%		10%	8%	
Further psychological assurance for low-risk women	Y	67%	67%	67%	4.63	82%	62%	16.6***	61%	71%	6.67*
	N	20%	13%	21%		13%	21%		26%	15%	
	DK	13%	20%	12%		5%	17%		13%	14%	
Earlier detection of chromosome abnormality	Y	64%	60%	65%	2.29	60%	65%	24.6***	57%	69%	4.48
	N	22%	20%	22%		37%	16%		25%	19%	
	DK	14%	20%	13%		3%	19%		18%	12%	
Earlier consideration of termination of pregnancy	Y	53%	49%	54%	2.30	52%	52%	10.5**	46%	58%	5.33
	N	33%	31%	34%		42%	30%		40%	28%	
	DK	14%	20%	12%		6%	18%		14%	14%	
Easier comprehension of test result	Y	50%	46%	51%	4.91	58%	47%	22.7***	44%	54%	6.53*
	N	28%	23%	30%		37%	25%		36%	23%	
	DK	22%	31%	19%		5%	28%		20%	23%	
Less maternal stress in case of termination of pregnancy	Y	38%	44%	36%	5.18	34%	39%	15.9***	28%	45%	10.4**
	N	42%	30%	45%		57%	36%		50%	36%	
	DK	20%	26%	19%		9%	25%		22%	19%	

Y = Yes; N = No; DK = Don’t Know. *p < .05; ** *p* < .01, *** *p* < .001

### Comparisons of NIPT with invasive prenatal diagnosis (IPD)

**Fig. 2:**
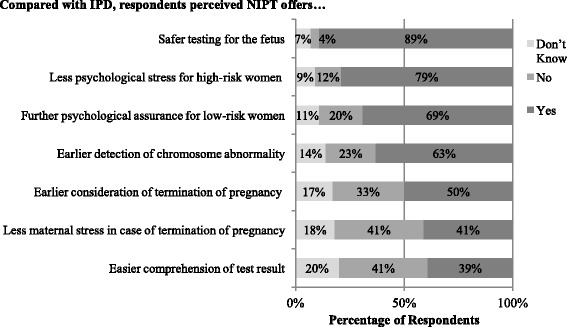
Comparison of Perceived Clinical Utility of NIPT with Invasive Prenatal Diagnosis (IPD)

Figure 2 shows respondents’ perceived clinical usefulness of NIPT compared with IPD. Respondents believed NIPT was likely to result in less psychological stress for high-risk women (79%) and more psychological assurance for low-risk women (69%). About 63% considered earlier detection of abnormalities as an advantage. Most respondents were unsure (41%) or did not believe that NIPT results were easier to understand than IPD. Fifty percent of the respondents believed NIPT enabled earlier consideration of TOP, although 33% were unsure. Respondents were divided on whether NIPT did (41%) or did not (41%) result in less maternal stress in case of TOP. Most respondents (89%) also believed NIPT provided safer testing for the fetus than IPD.

Overall, the responses show that respondents with university education had more formed views on the usefulness of NIPT (see Table 2), including its psychological benefits for high-risk women, comprehension of NIPT result, and procedural safety. Regarding profession, the pattern of significant differences was similar to that observed when comparing NIPT with FTS. Obstetricians were less likely than midwives to perceive that NIPT was as useful as IPD on psychological benefits. Also, professionals working in public hospitals were less likely than those in private hospitals to believe that NIPT was more useful than IPD for assuring low-risk women or resulting in less maternal stress in cases of TOP.

**Table 2:** Comparison of Perceived Clinical Utility of NIPT with IPD by Demographic Characteristics

		Total (*n* = 327)	High school (*n* = 70)	University (*n* = 257)	χ^2^	Obstetrician (*n* = 90)	Midwife (*n* = 237)	χ^2^	Public Sector (*n* = 136)	Private Sector (*n* = 191)	χ^2^
Safer testing for the fetus	Y	89%	77%	93%	14.2**	96%	87%	4.94	93%	86%	4.89
	N	4%	6%	4%		1%	5%		2%	6%	
	DK	7%	17%	3%		3%	8%		5%	8%	
Less physiological stress for high-risk women	Y	79%	69%	82%	13.6**	78%	80%	2.47	77%	81%	1.41
	N	12%	10%	12%		16%	10%		14%	10%	
	DK	9%	21%	6%		6%	10%		9%	9%	
Further psychological assurance for low-risk women	Y	69%	64%	71%	3.33	68%	70%	12.8**	61%	75%	7.89*
	N	20%	19%	20%		29%	16%		26%	15%	
	DK	11%	17%	9%		3%	14%		13%	10%	
Earlier detection of chromosome abnormality	Y	63%	59%	65%	8.03*	47%	70%	41.6***	60%	66%	1.46
	N	23%	17%	25%		48%	14%		25%	22%	
	DK	14%	24%	10%		5%	16%		15%	12%	
Earlier consideration of termination of pregnancy	Y	50%	49%	50%	7.78*	39%	54%	25.8***	40%	57%	8.28*
	N	33%	24%	36%		54%	26%		40%	29%	
	DK	17%	27%	14%		7%	20%		20%	14%	
Less maternal stress in case of termination of pregnancy	Y	41%	46%	39%	10.7**	32%	44%	27.9***	32%	47%	8.03*
	N	41%	26%	45%		62%	33%		48%	36%	
	DK	18%	28%	16%		6%	23%		20%	17%	
Easier comprehension of test result	Y	39%	49%	36%	21.6***	18%	46%	65.0***	30%	45%	11.6**
	N	41%	19%	47%		77%	28%		52%	34%	
	DK	20%	32%	17%		5%	26%		18%	21%	

Y = Yes, N = No, DK = Don’t Know

*p < .05; ** *p* < .01, *** *p* < .001

### Ethical concerns in the implementation of NIPT

**Fig. 3:**
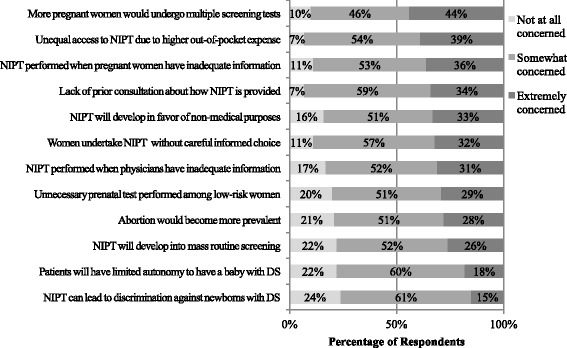
Ethical Concerns in the Implementation of NIPT. Numbers in the bar indicate the percentages of responses. From left to right, 5-point Likert scale is as follows: *light grey* = not at all concerned; *grey* = slightly-somewhat concerned; *dark grey* = moderately-extremely concerned

The three issues of most concern to respondents about NIPT were: more women would undergo multiple screening tests (44%); unequal access to NIPT due to higher out-of-pocket expense (39%); and NIPT being performed while women have inadequate information (36%, see Fig. 3). The two items that respondents were least concerned about (not at all or somewhat-slightly concerned) were related to social implications of NIPT, namely women would have limited autonomy to have a baby with DS (22%, 60%, respectively) and NIPT can lead to discrimination against new-borns with DS (24%, 61%, respectively). Obstetricians were more concerned than midwives about increasing uptake of NIPT without careful informed choice (29% vs. 42%, *p* = 0.05) and less concerned about increasing rates of abortion (33% vs. 14%, *p* < 0.001), women’s limited autonomy to have a baby with DS (22% vs. 8%, *p* < 0.001), and increasing discrimination of DS (18% vs. 6%, *p* < 0.001). Compared with professionals in the private sector, those in the public sector were more concerned about women undergo multiple screening tests (38% vs. 52%, *p* = 0.015), less concerned about increasing abortion (32% vs. 23%, *p* = 0.021), unnecessary uptake of NIPT among low-risk women (30% vs. 27%, *p* = 0.04), and use of NIPT for non-medical purposes (34% vs. 32%, *p* = 0.03) such as sex determination.

### Associations between clinical validity, perceived utility and ethical concerns

Table 3 shows multiple ordinal regressions of respondents’ views about the clinical usefulness of NIPT and its ethical implications on both univariate analysis and after adjusting for confounding factors. Respondents believed NIPT would result in less maternal stress in cases of TOP and were concerned about women undertaking multiple screening tests (AOR = 1.64). Respondents also believed NIPT was useful in the early detection of DS, but were concerned about increasing abortion rates for both FTS (AOR = 1.62) and IPD (AOR = 1.59). In addition, respondents believed NIPT could provide more psychological assurance for low-risk women than IPD, and had little concern about physicians’ lack of knowledge and skills in discussing NIPT with women (AOR = 0.63), lack of consultation with women (AOR = 0.51), and women undertaking the test without careful informed choice (AOR = 0.48). In contrast, respondents believed NIPT could provide a similar level of assurance as IPD, were concerned about the lack of consultation with women (AOR = 1.85) and women undertaking the test without careful informed choice (AOR = 2.16).

**Table 3:** Multiple Ordinal Regressions of Perceived Clinical Utility of NIPT for Ethical Concerns

		Abortion would become more prevalent	Patients will have limited autonomy to have a baby with Down syndrome	NIPT can lead to discrimination against newborns with Down syndrome	Testing performed when physicians have inadequate knowledge of NIPT	Lack of prior consultation about how NIPT is provided and performed	Increase in women’s undertaking NIPT without careful informed choice	More pregnant women would undergo multiple screening tests
		AOR^a^ (95%CI)	AOR^a^ (95%CI)	AOR^a^ (95%CI)	AOR^a^ (95%CI)	AOR^a^ (95%CI)	AOR^a^ (95%CI)	AOR^a^ (95%CI)
Comparison of NIPT with FTS								
Easier comprehension of test result	Y	1.04 (0.68--1.58)	1.12 (0.72–1.73)					
	N	0.72 (0.45--1.14)	0.72 (0.44–1.16)					
Earlier detection of chromosome abnormality	Y	1.62 (1.04--2.51)*						
	N	0.57 (0.34--0.97)*						
Earlier consideration of termination of pregnancy	Y							1.19 (0.78--1.82)
	N							1.45 (0.93--2.27)
Less maternal stress in case of termination of pregnancy	Y							0.94 (0.61--1.45)
	N							1.64 (1.06--2.54)*
Comparisons of NIPT with IPD								
Earlier detection of chromosome abnormality	Y	1.59 (1.02--2.48)*	1.10 (0.70--1.72)	0.81 (0.49--1.33)				
	N	0.56 (0.34--0.94)*	0.64 (0.38--1.08)	0.94 (0.53--1.69)				
Easier comprehension of test result	Y	1.11 (0.72--1.72)	0.91 (0.58--1.42)	0.82 (0.49--1.37)				
	N	0.51 (0.33--0.79)**	0.69 (0.43--1.10)	0.89 (0.52--1.51)				
Higher sensitivity	Y	0.98 (0.63–1.53)	1.37 (0.87--2.17)	0.92 (0.55--1.55)				
	N	0.59 (0.38--0.92)*	0.52 (0.32--0.84)**	0.75 (0.44--1.27)				
Further psychological assurance for low-risk women	Y				0.63 (0.40--0.99)*	0.51 (0.31--0.82)**	0.48 (0.29--0.81)**	
	N				1.34 (0.79--2.28)	1.85 (1.07--3.19)*	2.16 (1.21--3.87)**	
Earlier consideration of termination of pregnancy	Y							1.29 (0.82--2.05)
	N							1.13 (0.70--1.85)

Y = Yes, N = No, DK = Don’t Know (reference group)

*p < .05; ** *p* < .01, *** *p* < .001

^a^adjusted for demographics variables, including age, gender, religion, profession, highest education, place of work, years of experience

## Discussion

Overall, the obstetric professionals in our study believed that compared with tests in the current screening pathway in the public sector, the main usefulness of NIPT related to psychological benefits. They believed it would result in less stress in high-risk women and assurance in low-risk women, although not less stress in women considering TOP. The perceived psychological benefits may be due to the better sensitivity of NIPT [25], hence more acceptance of high-risk test result. However, despite perceived clinical usefulness of the test, the uncertainty and anxieties associated with unforeseen pregnancy outcomes relate to beliefs that TOP was likely to be stressful for women irrespective of the technology used. These findings reinforce that while newer, improved technology may be useful in alleviating some psychological implications of screening tests, obstetric professionals should provide pre-test information/counselling to ensure that women opting for screening tests for psychological assurance are aware of the psychological implications of a high-risk screening result, including decisions about TOP.

Diverse opinions about the clinical usefulness of NIPT by profession and healthcare sector suggest that clinical integration of the test to existing screening and diagnosis pathway remains in its infancy [14, 26]. Obstetricians had more formed views than midwives about the usefulness of NIPT, and the findings suggest that they believed NIPT was more useful than FTS, but not necessarily more useful than IPD. This is most likely due to NIPT being a highly accurate test, but is a screening test at best and not diagnostic. Furthermore, in relation to differences about ethical issues, obstetric professionals in the public sector were more concerned than those in the private sector about women undergoing multiple screening tests. This may be because they perceive few benefits in multiple screening tests and, therefore, may potentially withhold information about NIPT [27]. These findings suggest the need for guidelines in public hospitals about whether and how women should be informed about NIPT, and that any information provided clarifies that NIPT is a screening test.

Obstetric professionals in public hospitals reported more conservative views than those in the private sector, suggesting that NIPT was equivalent to FTS. Such a difference in views may result in a lack of referral by obstetric professionals in public setting to NIPT that is currently only available commercially. HK is a pioneering region for the development and implementation of NIPT in antenatal care, where pregnant women are exposed to information about NIPT through various media channels [28, 29]. Informing women identified at high-risk via FTS in the public sector about NIPT could ensure that they receive similar information to women in the private sector, although consideration should also be given to the ethical implications of raising awareness about a test in women in the public sector unable to afford it. Therefore, there is a need for debate and the development of guidelines for obstetric professionals in public hospitals on whether NIPT should be an option presented to high-risk women during counselling, and the extent to which this relates to women’s autonomous decision-making.

Our findings are important for informing current debates globally as to whether NIPT should be introduced as a primary screening test. Obstetric professionals believed the use of NIPT would enable early detection of the fetus’s condition, which was important for reassuring women, and because early detection may result in women experiencing less physically/psychological burden due to earlier consideration of selective abortion [28]. These findings suggest obstetric professionals’ preference for earlier screening and diagnostic tests to enable early selective abortion. However, any policy/clinical consideration of implementing NIPT as early as possible along the antenatal screening pathway, say as a primary screening test, would also need to take into account the implications of using an earlier IPD procedure, i.e. CVS at 10-13th weeks of gestation. This is because CVS has an increased risk for fetal loss compared with amniocentesis [30, 31]. Furthermore, amniocentesis is preferable over CVS to avoid duplicating the original placental mosaicism that resulted in primary NIPT screening result: both CVS and NIPT may be then misleading when there is confined placental mosaicism [32]. The extent to which obstetric professionals and pregnant women are aware of the increased risk of CVS as an earlier diagnostic test (to complement the earlier NIPT test) in comparison to amniocentesis is not known, and the extent to which this increased risk would influence obstetric professionals’ or women’s preferences for the type of screening test, subsequent diagnostic test, or timing of these test needs further research.

Half of the respondents in the present study believed that NIPT results were not easier for them to understand than FTS. Reporting formats of NIPT results vary across laboratories and most in HK present results in categories (i.e., either yes/no or positive/negative). Perhaps obstetric providers not only need to understand the mechanism of how cff-DNA sequencing technology yields higher analytical validity yet not performed as confirmatory testing [33], but also need to be aware of the differences between information reported by individual NIPT laboratories and the potential validity of patients’ result. Currently, the present fetal fraction is not universally reported, where obstetric professionals must accept NIPT results at a face value and assume that there was adequate DNA of fetal origins present [34]. A platform supporting education initiatives for obstetric professionals is essential, firstly, to ensure that they understand various ways in which laboratories may report NIPT results, understand their implications for pregnant women, and how best to communicate these results to women and secondly to ensure women are fully informed about the test.

Similar to our study, recent studies in the UK and US report favourable attitudes towards NIPT over conventional DS screening among obstetric professionals mainly due to clinical advantages [35, 36]. It is important to note that the obstetric professionals in our study, who believed NIPT was a more useful test than FTS and IPD, expressed less ethical concerns than those who were uncertain about NIPT test characteristics. These findings suggest the need for a balanced approach to training obstetric professionals about NIPT. That is, while better knowledge of NIPT could alleviate obstetric professionals’ uncertain ethical concerns, they need to be aware of women’s need for pre- and post-test consultation on NIPT ensuring that women are making informed reproductive choices. Prenatal screening providers’ limited familiarity and experience with NIPT and limited resources to assist with counselling were common barriers encountered when providing women with counselling about NIPT [37], highlighting the unmet services needs in providing pre- and post-test information on the clinical usefulness and limitations of NIPT [38–40]. Practice guideline for obstetric professionals in both private and public sectors should set out the need for the provision of pre- and post-NIPT information needs to ensure the provision of a service that meets women’s need for information.

The finding of positive associations of further psychological assurance in low-risk women with ethical concerns about physician competency (inadequate knowledge of NIPT and lack of prior consultation about how NIPT) and ‘increase of undertaking NIPT without informed choice’ further suggest the need for debate and development of guidelines on whether and how NIPT should be mentioned by obstetric professionals on the current DS screening pathway in public hospitals. This is particularly important in light of our previous research showing that women screened low-risk following FTS preferred to use NIPT as means of “buying another insurance” to confirm a previously received negative test result in pregnancy care [28]. Women’s preferences for NIPT may be affected by how and what information they receive from various sources ranging from lay-media, social influence, and professional opinions [28, 41]. Unlike Western contexts, there is a need for post-test counselling for low-risk women to enable better decision-making about further tests, because they are likely to undertake multiple DS screening tests, both FTS and NIPT, for psychological assurance despite the absence of an indication of a fetal anomaly [28].

The perceived usefulness compared with FTS and IPD was not associated with the item on the ethical concerns addressing public health implications of NIPT, including an increase of mass routine screening, unequal access to NIPT due to high cost, and development of NIPT for non-medical purposes. It appears that these concerns are independent of how NIPT is perceived in comparison with FTS and IPD. Possible explanations would be, as stated earlier, HK provides universal DS screening for women irrespective of age and the scale-up rates of FTS were already high, over 90% [21]. Clinical adoption of NIPT might not affect the rate of routine screening in HK. The concerns about a high-cost private test and non-medical use of NIPT have little to do with a comparison of usefulness with existing prenatal testing for DS.

This was a cross-sectional study in a specific population, which inevitably reduces the confidence with which the findings can be generalised. However, the findings are likely to be generalizable to other countries with similar clinical contexts, such as those offering government funded antenatal screening programmes for DS and likely to consider the implementation of NIPT within this screening pathway. Further research capturing the views of obstetric professionals in other Eastern countries could enhance our understanding of the ethical and social implication of NIPT in this part of the world. The lower responses rate among obstetricians compared to midwives may have been due to the different recruitment strategies adopted. Midwives were invited in person by a researcher, while obstetricians were approached via a postal invitation because of our assumption of their comparatively limited time during work hours to discuss research participation. A more personal approach to recruiting may have led to a better participation rate of obstetricians. Qualitative research approaches could provide a more in-depth understanding of the reasons for the views expressed by the obstetric professionals in this study. Furthermore, research is needed with a wider range of stakeholders, including the views of service users and individuals responsible at a policy level for commissioning, regulating and developing guidelines for the use of genetic technologies. Nevertheless, our study highlights important points that could inform debates at policy level about the implementation of NIPT within public services in HK and other countries.

## Conclusions

With the trend for rising maternal age at childbirth and lower fertility rates in many parts of the world, more pregnant women are deemed high-risk for DS and, therefore, likely to want to access NIPT as a preventive measure. Meanwhile, more fetal conditions are likely to be detected by NIPT using cff-DNA sequencing in practices. Our findings highlight the educational and training needs of obstetric professionals, the need for policy debates on the potential implementation of NIPT within public services, and an urgent need for the development of a standard care guideline on how to ensure pregnant women who use public antenatal services are informed about commercially available more advanced prenatal technology.

## Abbreviations

cff-DNA: Cell-free DNA
CVS: Chorionic villus sampling
DS: Down syndrome
ELSI: Ethical, legal and social implication
FPR: False-positive rate
FTS: First-trimester combined screening
HK: Hong Kong
IPD: Invasive prenatal diagnosis
NIPT: Non-invasive prenatal testing
PPV: Positive predictive value
TOP: Termination of pregnancy
